# The Global States and Hotspots of ERAS Research From 2000 to 2020: A Bibliometric and Visualized Study

**DOI:** 10.3389/fsurg.2022.811023

**Published:** 2022-03-09

**Authors:** Shengjie Su, Tonghao Wang, Ruiyuan Wei, Xiaowu Jia, Qiang Lin, Minghua Bai

**Affiliations:** First Department of Orthopaedics and Traumatology, Baoji Traditional Chinese Medicine Hospital, Baoji Orthopaedic Hospital, Baoji Institute of Traditional Chinese Medicine, Baoji, China

**Keywords:** ERAS, fast-track, bibliometric, visualized study, surgery, hotspots

## Abstract

**Background:**

Enhanced recovery after surgery (ERAS) protocol has been implemented in surgeries for more than 20 years, this study investigated the global states and hotspots of ERAS research.

**Methods:**

Based on the Web of Science database, a bibliometric and visualized study of original ERAS research from 2000 to 2020 was performed, including the trends of publications and citations; distribution of countries, authors, institutions, sources; study design, level of evidence, served surgeries and surgical disciplines. Hotspots were revealed by research interests and keywords.

**Results:**

Within the field of original ERAS research, there was a rising trend in annual publications and citations. The USA was the greatest contributor. Kehlet, H, University of Copenhagen were the most influential author and institution, respectively. British Journal of Surgery and Annals of Surgery were the most cited journals. Though there were more prospective designs, more than half of the studies presented level IV evidence and had fewer citations and citation densities compared to that of level II and level III. ERAS protocol was overwhelmingly implemented in colorectal surgeries. Most studies focused on elements of ERAS, the top three research interests were “length of stay,” “pain management,” and “complications.” In recent years, bariatric surgery, compliance with ERAS, and feasibility in the elderly were new hotspots.

**Conclusion:**

Revealing the global states and hotspots can help researchers better understand the trends in ERAS research. The USA was the greatest contributor to ERAS research. Kehlet, H, was the most influential author in the field. Bariatric surgery, compliance with ERAS, and feasibility in the elderly represent the new trend of ERAS research. Most of the ERAS research had a low evidence levels, studies with high-level evidence are still required in this field.

## Introduction

In 2001, the Enhanced Recovery After Surgery (ERAS) Study Group was established in Europe, then they published the first consensus for colonic resection in 2005 (1). Before that, the concept of fast-track surgery had been used for several years. The earliest attempt of accelerated recovery after surgery can be traced back to 1990, Krohn BG et al. reported the experience of rapid recovery for open heart operations for the first time (2). In 1994, Engelman RM (3) described the first fast-track recovery protocol for patients undergoing cardiopulmonary bypass surgery, followed by similar research on multimodal management protocols for colonic surgery (4) and open sigmoidectomy (5). Though the ERAS concept has been widely adopted around the world, and the ERAS Group wanted to emphasize the importance of quality rather than speed of recovery (6), many researchers still use the concept of “fast-track surgery.” Meanwhile, as an important part of ERAS protocol, fast-track anesthesia has been promoted greatly by the progress of ERAS. To our knowledge, few studies have systematically analyzed the global states and hotspots of original ERAS research, we performed a 20-year bibliometric and visualized study to help researchers better understand the trends in ERAS research.

## Methods

### Literature Search

This study was based on the Web of Science (WOS) database. The following search strategy was used: “TS=enhanced recovery after surgery” or “TS=fast-track surgery,” all subdatabases were retrieved, including Web of Science Core Collection, SciELO Citation Index, KCI-Korean Journal Database, MEDLINE®, BIOSIS Previews, and Russian Science Citation Index. The publication year was restricted from 2000 to 2020, regardless of language.

### Article Screening

**Figure 1:**
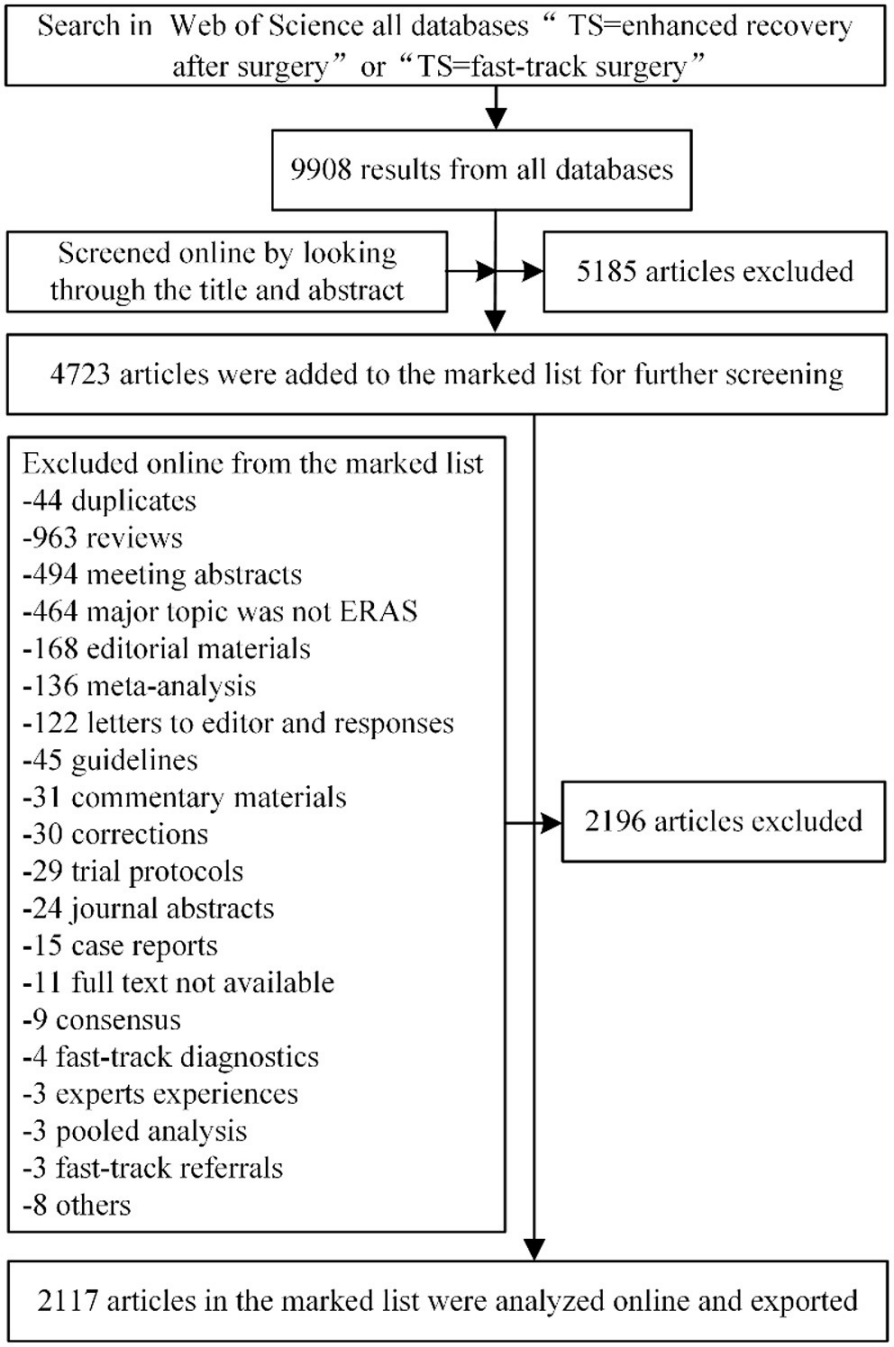
Flowchart of article screening.

Literature were screened online on the WOS website, articles that contained “enhanced recovery” or “fast track” in the title or abstract were filtered preliminarily. The further exclusion criteria were as follows: only original research were included, review (including systemic review, meta-analysis, and pooled analysis), guidelines, case reports, expert experiences, consensus, meeting abstract, editorial materials, letters and responses, commentaries, corrections, trial protocols, position papers, animal studies, suggestions, special articles, book chapters, highlights, journal abstracts were excluded. Besides, fast-track diagnostics, fast-track referrals systems, and articles that did not focus on ERAS were excluded. Finally, 2,117 articles were included for bibliometric analysis (Figure 1). Articles were imported to literature management software Endnote X9 (Clarivate Analytics, Philadelphia, PA, USA), Microsoft Excel 2019 (Microsoft Corp. Redmond, WA, USA), Vosviewer 1.6.16 (Leiden University, Leiden, The Netherlands), and Citespace 5.7 (Drexel University, Philadelphia, PA, USA) for analysis.

### Bibliometric Analysis

The annual number of publications and citations was counted. The citation density was defined as the citation per year (total citations/years since published). The country distribution was determined by corresponding authors, if there were more than one corresponding author, the last corresponding author was selected.

The level of evidence was graded following the Oxford Center for Evidence-Based Medicine (OCEM) 2011 Levels of Evidence system (7). Two authors looked through the full article texts and graded the level independently, divergences were resolved by discussion. Comparison of citations and citation density was performed among different levels of evidence. Names of surgery in each article were distracted and divided into different surgical disciplines. Research interests were summarized in the full texts.

### Visualization

Vosviewer and Citespace were used for visualized analysis, including bibliographic coupling analysis of authors, institutions, and journals; co-cited analysis of cited authors in the reference lists; co-occurrence analysis of author keywords. For author keywords, an overlay visualization map weighed by average published year was shown in Vosviewer, burst detection was further performed by Citespace to reveal the hotspots by years.

### Statistical Analysis

SPSS 24.0 (IBM Corporation, Armonk, NY, USA) was used for statistical analysis. The distribution of continuous variables was checked by using the sing-sample Kolmogorov-Smirnov test, non-normally distributed data were presented as median (interquartile range, *IQR*). The homogeneity tests showed uneven variance among multiple samples, thus the analysis of variance was not suitable, comparison between multiple variables was performed by the Kruskal-Wallis H test, which was used for multiple comparisons among independent variables. The Kolmogorov-Smirnov test showed that the years and publications, citations are non-normal distributed data, so the correlation between the year and the publications; year and citations was tested by the Spearman test. The Kappa consistency test was used to determine the consistency between the two authors in the grading of the level of evidence. The significance level was defined as 0.05.

## Results

### Publications and Citations

Of the 2117 articles, 1720 were from Web of Science Core Collection Database, 351 were from MEDLINE® Database, 19 were from Russian Science Citation Index Database, 12 were from BIOSIS Previews Database, 9 were from SciELO Citation Index Database, and 6 were from KCI-Korean Journal Database. The majority of the articles were written in English (90.55%), followed by Chinese (2.78%), Russian (1.51%), German (1.36%), and Spanish (1.18%), the remaining 15 Languages each counted <1%. By March 18, 2021, the 2117 articles had a total h-index of 83, total citation of 38,114 (29,243 without self-citations). The citations of each article ranged from 0 to 546 times, the median citation was 6 (1, 21), the median citation density was 1.67 (0.36, 3.96).

**Figure 2:**
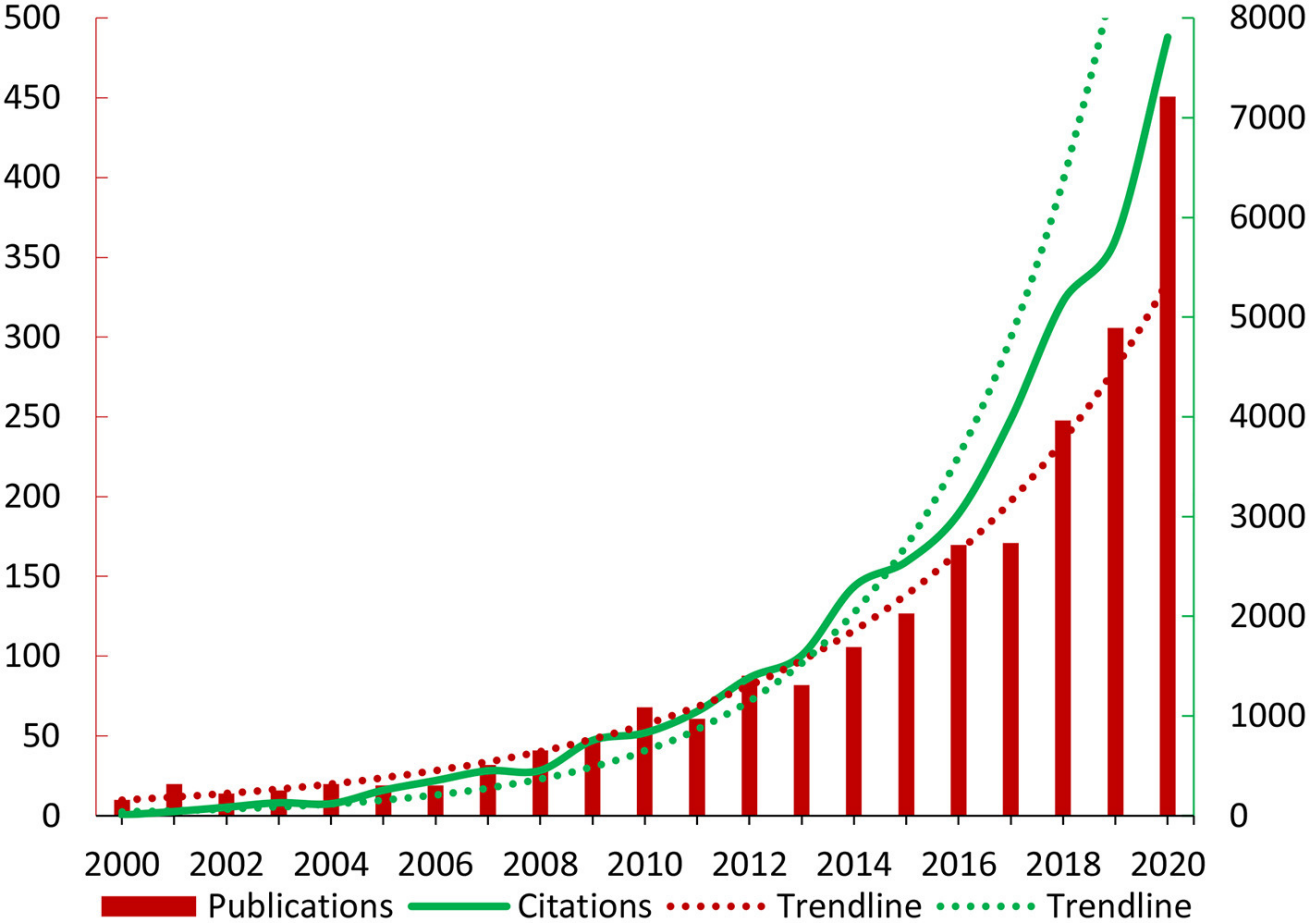
The trend in publications and citations.

From 2000 to 2020, there was an annually rising trend of publications and citations (Figure 2). The number of citations has been increasing quickly since 2014. Spearman correlation test revealed a strong positive correlation between year and citations (ρ = 0.99, *P* < 0.001); year and publications (ρ = 0.97, *P* < 0.001).

### Country Distribution

**Figure 3:**
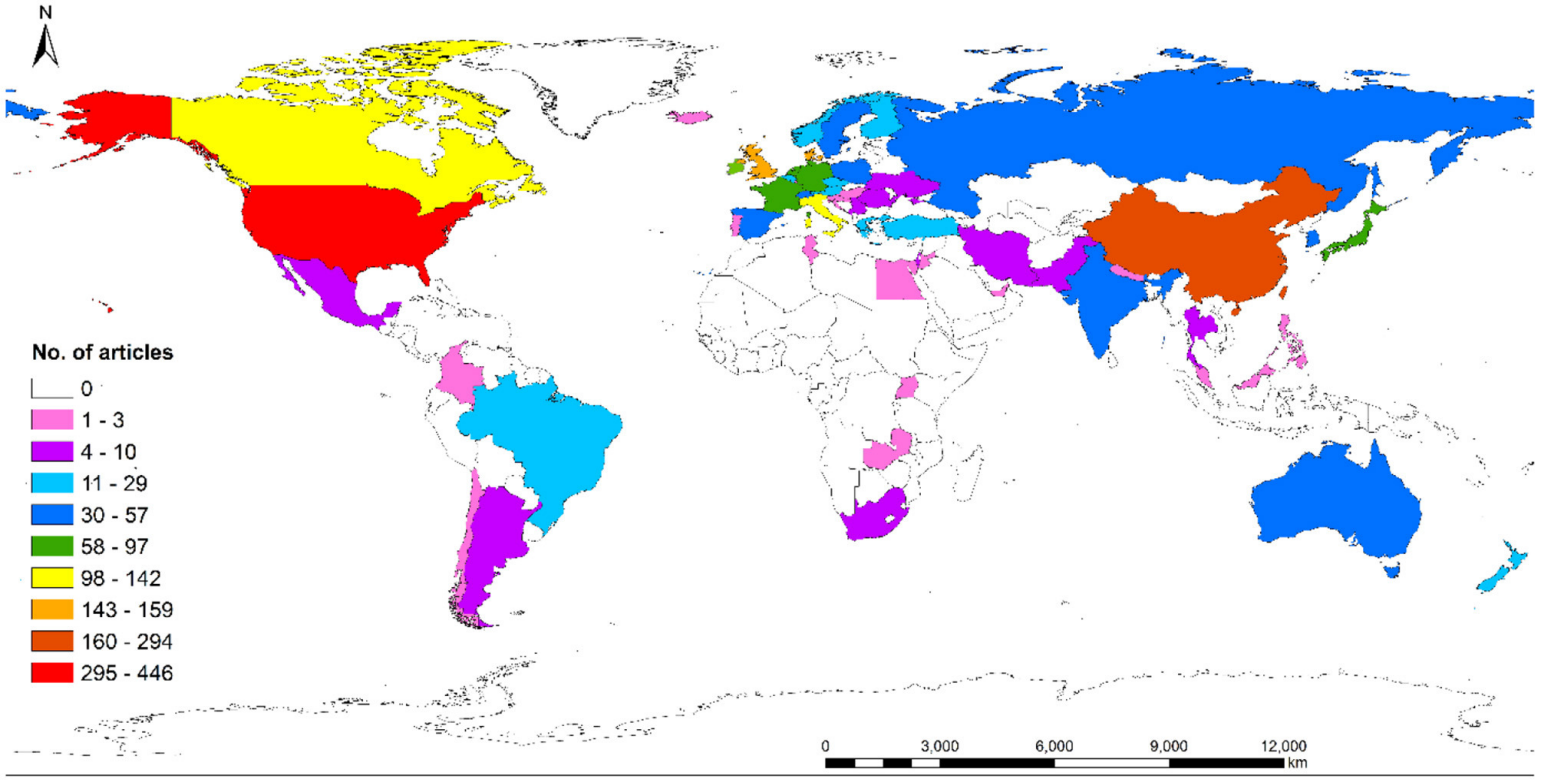
Global distribution of ERAS research.

The 2,117 articles were from 56 countries (Figure 3), the USA contributed the greatest number of articles, with 446 articles (21.07%) and 6,940 citations, the h-index was 42. The second-largest contributor was China, with 293 articles (13.84%) and 2,460 citations, the h-index was 26. The third was England, with 159 articles (7.51%) and 4,341 citations, the h-index was 34. The top 10 countries with the greatest number of publications were listed in Table 1.

**Table 1:** Top 10 countries with the greatest number of publications.

**Country**	**Publications**	**Citations**	**Citations per item**	**h-index**
USA	446	6,940	15.56	42
China	293	2,460	8.39	26
England	159	4,341	27.30	34
Denmark	143	5,752	40.22	38
Italy	112	1,483	13.24	23
Canada	98	2,083	21.25	29
Germany	85	2,045	24.06	25
Japan	78	705	9.04	15
Netherlands	74	3,213	43.42	28
France	75	523	6.97	13

### Authors and Institutions

**Figure 4:**
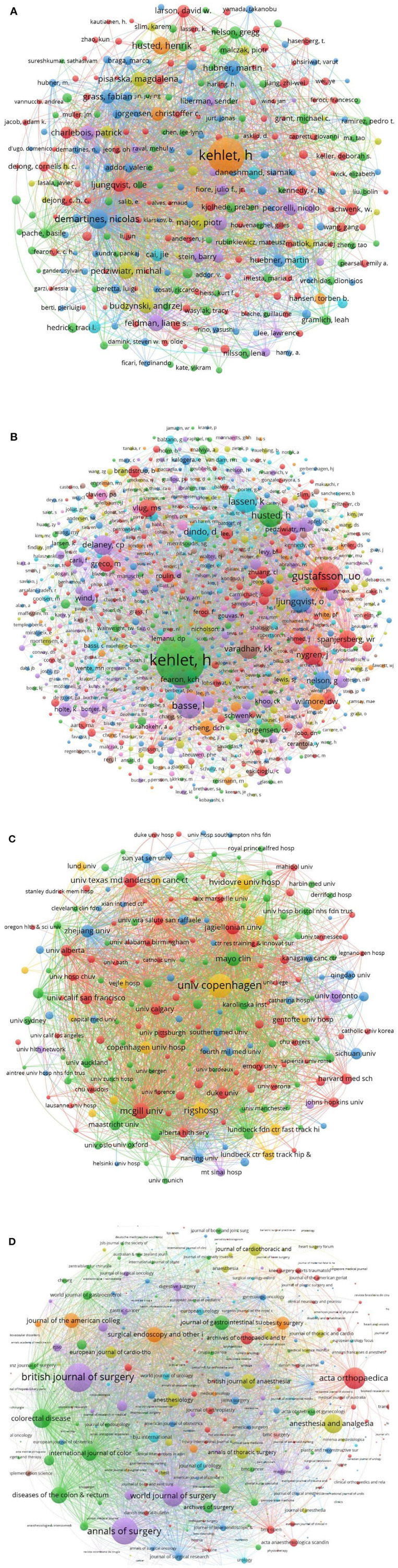
Visualized analysis of authors, cited authors, institutions, journals. **(A)** Bibliographic coupling analysis of authors. **(B)** Co-citation analysis of cited authors. **(C)** Bibliographic coupling analysis of institutions. **(D)** Journals.

The author with the greatest number of publications and citations was Kehlet, Henrik, with 98 articles and 5,275 citations, the h-index was 38. The visualized analysis showed that the total link strength was 91,772. The second was Demartines, Nicolas, with 34 articles and 1,106 citations, the h-index was 15, the total link strength was 114,763. Followed by Huebner, Martin, with 34 articles and 826 citations, the h-index was 15, the total link strength was 46,188 (Figure 4A). The top 10 authors with the greatest number of publications were listed in Table 2.

**Table 2:** Top 10 authors with the greatest number of publications.

**Author**	**Affiliation**	**Publications**	**Citations**	**h-index**
Kehlet, Henrik	University of Copenhagen	98	5,275	38
Demartines, Nicolas	University of Lausanne	34	1,106	15
Huebner, Martin	University of Lausanne	34	826	15
Husted, Henrik	University of Copenhagen	28	1,573	19
Jorgensen, Christoffer Calov	University of Copenhagen	27	656	15
Feldman, Liane S.	McGill University	22	391	11
Ljungqvist, Olle	Orebro University	21	2,114	17
Carli, Francesco	McGill University	18	538	13
Pedziwiatr, Michal	Jagiellonian University	20	459	14
Schwenk, Wolfgang	Klin Allgemein	20	536	13

Co-citation analysis of cited authors showed that Kehlet, Henrik was the top-cited author in the reference lists, who was cited by 1,541 times and the total link strength was 22,638. Followed by Gustafsson, Ulf O, cited by 508 times and the total link strength was 8,980. The third was Basse, Line Hollesen, cited by 414 articles and total link strength was 7,083 (Figure 4B). The top 10 most cited authors were listed in Table 3.

**Table 3:** Top 10 most-cited authors in the reference lists.

**Author**	**Affiliation**	**Cited times**	**Total link strength**
Kehlet, Henrik	University of Copenhagen	1,541	3,331
Gustafsson, Ulf O	University of Lausanne	508	1,402
Basse, Line Hollesen	Novo Nordisk	414	1,452
Husted, Henrik	University of Copenhagen	346	541
Lassen, Kristoffer	National Hospital Norway	329	1,051
Ljungqvist, Olle	McGill University	287	737
Dindo, Daniel	Hirslanden Med Ctr	271	718
Delaney, Conor P.	Dana-Farber Cancer Institute	249	1,032
Nygren, Jonas	Karolinska Institutet	243	923
Varadhan, Krishna K.	Nottingham University Hospital NHS Trust	235	821

The institution with the greatest number of publications and citations was the University of Copenhagen, with 117 articles and 5,939 citations, and an h-index of 38, the total link strength was 63,857. Followed by the Rigshospitalet, with 94 articles and 3,823 citations, and an h-index of 34, the total link strength was 30,753 (Figure 4C). The top 10 institutions with the greatest number of publications were listed in Table 4. The top 10 articles with the greatest number of citations were shown in Table 5.

**Table 4:** Top 10 institutions with the greatest number of publications.

**Institution**	**Country**	**Publications**	**Citations**	**h-index**
University of Copenhagen	Denmark	117	5,939	38
Rigshospitalet	Denmark	94	3,823	34
University of Lausanne	Switzerland	43	956	17
University of Texas system	USA	40	868	15
Lundbeckfonden	Denmark	36	879	18
Aarhus University	Denmark	34	881	17
McGill University	Canada	32	633	14
University of California System	USA	32	562	14
Mayo Clinic	USA	27	663	15
Humboldt University of Berlin	Germany	26	637	15

**Table 5:** The top 10 most cited articles.

**Author/year**	**Title**	**Citations**
Basse et al. (8)	A clinical pathway to accelerate recovery after colonic resection	545
Vlug et al. (9)	Laparoscopy in Combination with Fast Track Multimodal Management is the Best Perioperative Strategy in Patients Undergoing Colonic Surgery A Randomized Clinical Trial (LAFA-study)	508
Gustafsson et al. (10)	Adherence to the Enhanced Recovery After Surgery Protocol and Outcomes After Colorectal Cancer Surgery	409
Basse et al. (11)	Functional recovery after open vs. laparoscopic colonic resection - A randomized, blinded study	340
Husted et al. (12)	Predictors of length of stay and patient satisfaction after hip and knee replacement surgery - Fast-track experience in 712 patients	332
Maessen et al. (13)	A protocol is not enough to implement an enhanced recovery programme for colorectal resection	317
Basse et al. (14)	Colonic surgery with accelerated rehabilitation or conventional care	313
Currie et al. (15)	The Impact of Enhanced Recovery Protocol Compliance on Elective Colorectal Cancer Resection Results From an International Registry	297
King et al. (16)	Randomized clinical trial comparing laparoscopic and open surgery for colorectal cancer within an enhanced recovery programme	267
Delaney (17)	“Fast track” postoperative management protocol for patients with high co-morbidity undergoing complex abdominal and pelvic colorectal surgery	261

### Journals

These articles were published in 597 journals, the journal with the greatest number of publications was Surgical Endoscopy and Other Interventional Techniques, with 54 articles, followed by the World Journal of Surgery, and Colorectal Disease. When weighted by citations, the visualized analysis showed that the British Journal of Surgery and Annals of Surgery had the greatest number of citations, followed by Acta Orthopaedica (Figure 4D). The top 10 most often published journals were listed in Table 6.

**Table 6:** Top 10 most often published journals.

**Journal**	**Country**	**Publications**	**Citations**	**IF (2020)**
Surgical Endoscopy and Other Interventional Techniques	USA	54	816	4.584
World Journal of Surgery	USA	48	1,574	3.352
Colorectal Disease	England	42	1,234	3.788
International Journal of Colorectal Disease	Germany	42	781	2.571
Acta Orthopaedica	England	34	1,555	3.717
Obesity Surgery	Canada	32	571	4.129
British Journal of Surgery	England	31	2,751	6.939
Annals of Surgery	USA	31	2,459	12.969
Journal of Cardiothoracic and Vascular Anesthesia	USA	31	568	2.628
Diseases of the Colon & Rectum	USA	26	949	4.785

*IF, impact factor*.

### Study Design and Level of Evidence

There were 692 articles (32.68%) with prospective study design, 549 articles (25.93%) with retrospective design (including retrospective analysis of prospectively collected data), and 22 articles with combined design (1.03%), study designs were not mentioned in the remain 854 articles. In terms of levels of evidence, 407 articles presented level II evidence (19.23%), 473 articles presented level III evidence (22.34%), while more than half of them presented level IV evidence (55.69%). The remaining 58 articles could not be graded according to the OCEM system. The agreement between the two authors was excellent (*kappa* = 0.97, *P* < 0.001). Among them, 1,769 articles (83.56%) were therapeutic analysis, 155 articles were prognostic analysis, 101 articles were surveys, 31 articles were cost-effective analysis, 55 articles were other designs.

**Figure 5:**
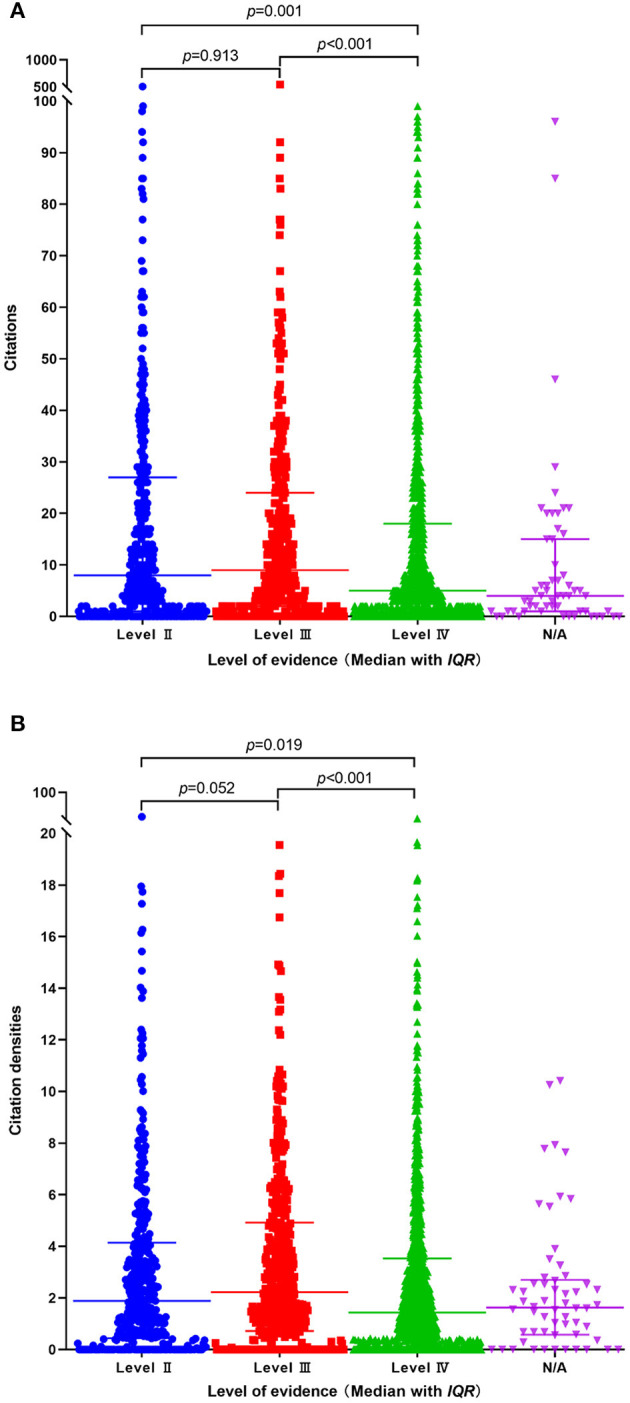
Comparison of citations and citation density between different levels of evidence. **(A)** citations. **(B)** citation density.

The Kruskal-Wallis H test showed that there was a significant difference in citations between level II and level IV studies (Z = 3.37, *P* = 0.001); level III and level IV studies (Z = 3.70, *P* < 0.001). While there was no significant difference in citations between level II and level III studies (Z = −0.11, *P* = 0.913) (Figure 5A). In terms of citation density, there was a significant difference between level II and level IV studies (Z = 2.34, *P* = 0.019); level III and level IV studies (Z = 4.89, *P* < 0.001), while there was no significant difference between level II and level III studies (Z = −1.94, *P* = 0.052) (Figure 5B).

### Research Interests and Surgeries

**Figure 6:**
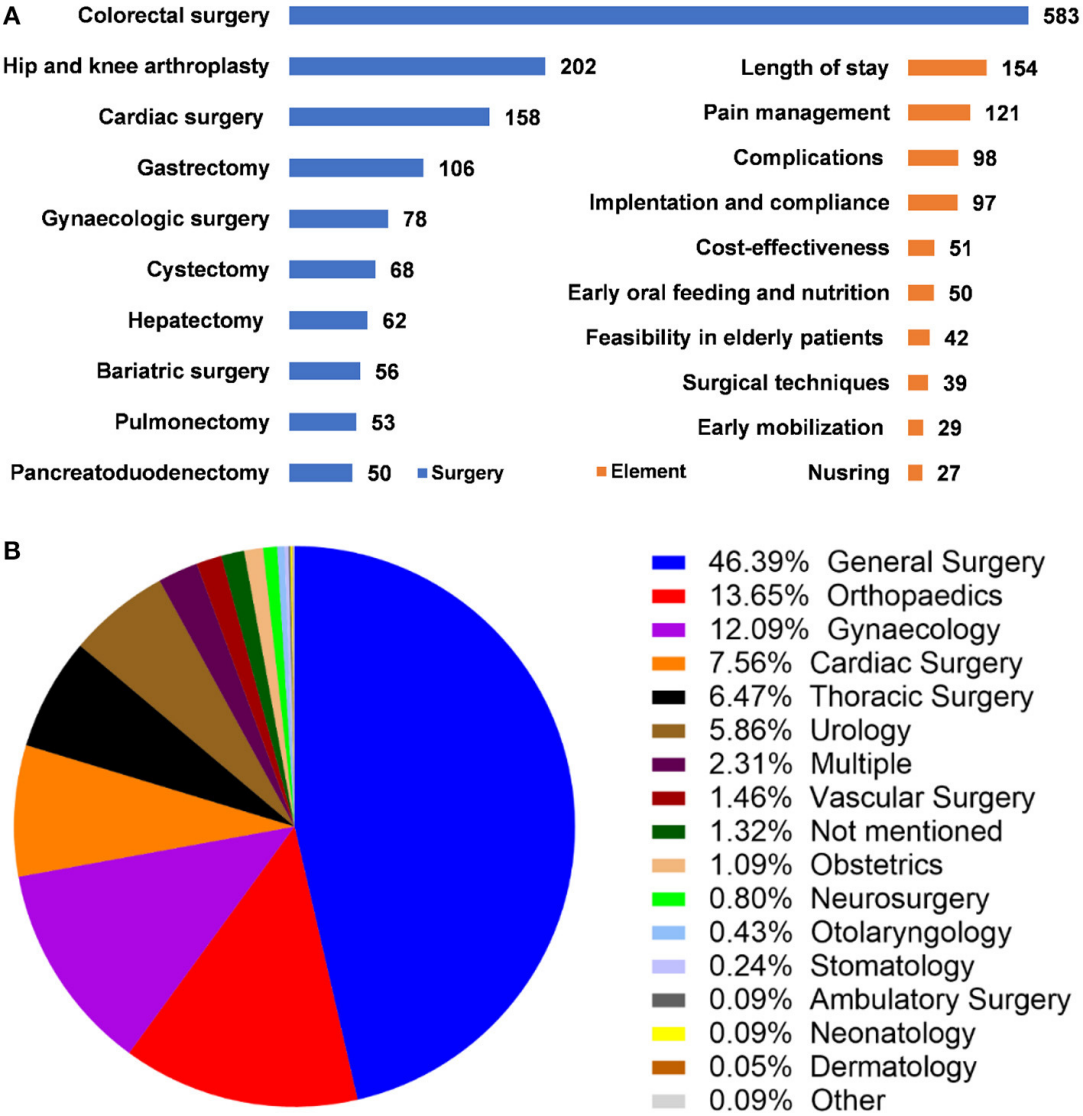
Surgeries, most focused elements, and surgical disciplines of ERAS research. **(A)** Top 10 surgeries and most focused ERAS elements. **(B)** Surgical disciplines that implement ERAS protocol.

The ERAS was most often implemented in colorectal surgeries, with 583 articles, followed by hip and knee arthroplasty (202 articles), and cardiac surgery (106 articles). The top three most focused elements were “length of stay,” “pain management,” and “complications.” The top 10 surgeries and most focused elements were shown in (Figure 6A). When divided into the surgical disciplines, the Department of General Surgery, Orthopedics, Gynecology, Cardiac Surgery, Thoracic Surgery were the top 5 disciplines that implemented most ERAS protocols (Figure 6B).

### Keywords

**Figure 7:**
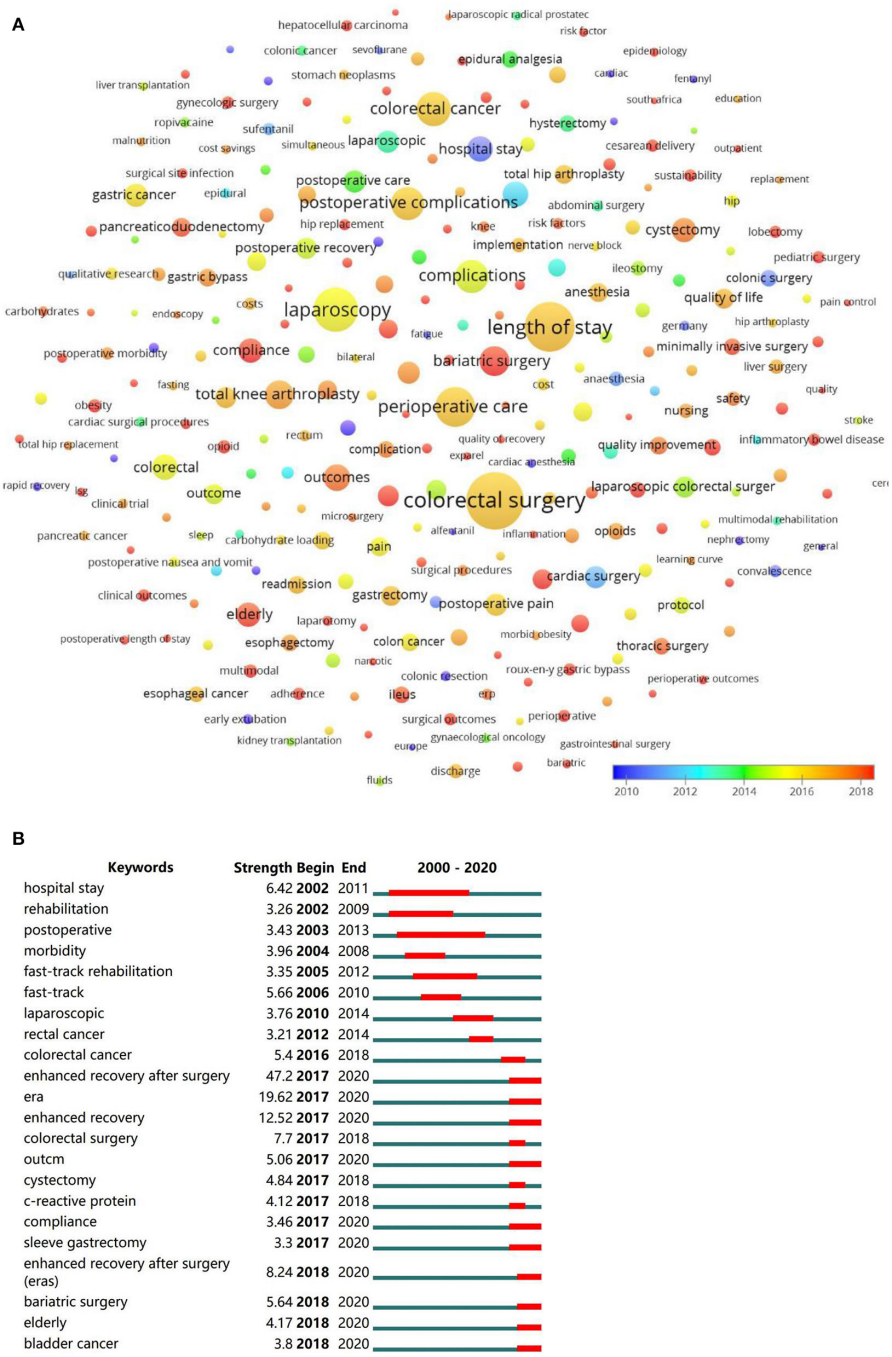
Visualized analysis and burst detection of author keywords. **(A)** Co-occurrence analysis of author keywords, weighed by average occurred year. **(B)** The burst detection of keywords.

Except for the theme words “ERAS” and “fast-track surgery,” co-occurrence analysis showed that “colorectal surgery” was the most frequently occurring keyword, with an occurrence of 146 times and total link strength of 211. Followed by “length of stay” (occurrence 109, total link strength 198), “laparoscopy” (occurrence 87, total link strength 156), “perioperative care” (occurrence 69, total link strength 116), “complications” (occurrence 49, total link strength 105). When ranked by year of occurrence, the top five most frequently used keywords occurred around 2016. “bariatric surgery,” “bladder cancer,” “cystectomy,” “Compliance,” “elderly” frequently occurred in recent years (Figure 7A).

In the burst detection, keywords were classified into several clusters, cluster “enhanced recovery after surgery” burst since 2017, “fast-track surgery” burst during 2005–2012, “colorectal surgery” burst during 2016–2018. “Cystectomy,” “bladder cancer,” “bariatric surgery,” “compliance,” “elderly” were keywords burst in recent years (Figure 7B).

## Discussion

This bibliometric and visualized study identified 20-year original articles in the field of ERAS research and analyzed their characteristics, revealing the global states and hotspots that can help researchers better understand the trends of ERAS research.

The academic impacts of researchers, institutions, and countries in a certain field are usually measured by the publications and citations, however, some limitations must be emphasized. The publication and citation counts can be influenced by the selection of databases, search strategy, and personal bias. A previous bibliometric analysis found that the Web of Science and Scopus exported very similar articles but different citations (18). Besides, the number of citations can be influenced by literature age, some newly published articles had low citations despite their high scientific values. Except for publications and citations, reputations, peer reviews, and impact factors should also be considered (19). Another phenomenon is that articles from inventors tended to be frequently cited, even though the techniques or concepts have been modified or abandoned. Despite these controversies, citations are still the most widely used tool in bibliometric analysis (19). In the present study, the WOS database was retrieved and visualized networks of authors, institutions, and sources based on the publications and citations were presented.

The USA was the largest contributor to original ERAS research, with the highest h-index of 42. Though China published the second large amount of publications, the number of citations was small compared to other countries, indicating a requirement of improved study qualities. When weighed by h-index, Denmark was the second influential country in ERAS research, the h-index was just second to that of the USA. The third country with the greatest number of publications and citations was England, with an h-index of 34. It was worth noting that the Netherlands owned the greatest number of citations per item, despite the relatively small number of publications. As was mentioned above, the evaluation of the impact of institutions and countries can be greatly influenced by some productive authors, in this study, most contributions of Denmark were from the University of Copenhagen and its affiliated hospitals.

The high-impact authors and institutions were revealed by the visualized analysis. The bibliographic coupling analysis showed a network of authors and institutions with the greatest number of publications and citations, while the co-citation analysis presented the most frequently cited authors in the reference lists. Keheht H, a surgeon from Rigshospitalet, University of Copenhagen, was the author with the greatest number of publications and citations, as well as the most frequently cited author. He was a member of the first ERAS Study Group and advocated the use of epidural anesthesia for postoperative pain control (4). It should be noticed that the contribution of Rigshospitalet belonged to the University of Copenhagen though it was calculated separately in the visualized network. The second most influential institution was the University of Lausanne, Demartines, Nicolas, and Huebner, Martin were the most productive authors, Gustafsson, Ulf O was the most cited author in this institution.

The top-cited article was from Basse et al. who perfumed a multimodal rehabilitation program of 48-h postoperative stay for patients undergoing colonic resection (8). The article was published in the Annals of Surgery in 2000 and was cited by 547 times in the WOS database. The second most cited article was a multicentre, randomized clinical trial that compared the laparoscopic and open resection of colon cancer combined with fast-track care (9). The article belonged to Vulg, MS et al. and was published in the Annals of Surgery in 2011, cited by 473 times. The third most cited article belonged to Gustafsson et al. (10), who found that improved adherence to ERAS protocol significantly improved the outcomes of patients undergoing colorectal surgery. It was published in the Archives of Surgery in 2011 and cited by 409 times. Among the top 10 most cited articles, all of the ERAS protocols were implemented in colorectal surgery except for one in hip and knee arthroplasty. Most of the highly-cited papers were from the British Journal of Surgery and Annals of Surgery, indicating their high reputations within the ERAS research. Revealing the high-impact sources also helps researchers select journals during submission.

In the present study, the OCEM 2011 level of evidence system was used (7). Since the systemic review and meta-analyses were not included, there was no Level I evidence. We found that randomized controlled trials counted only 19.46% among the study designs, though there were more prospective designs, more than half of the studies were case serious or case-control studies and presented level IV evidence. Retrospective analysis of prospectively collected data was considered as retrospective designs, besides, there were a few studies with combined designs. Considering the influence of literature age on citations, we calculated the citation density to weigh the average citations by year. We found that articles with level IV evidence had fewer citations and citation densities compared to that of level II and level III, respectively. While the number of citations and citation densities were comparable between level II and level III studies, suggesting that articles with level IV evidence were less likely to be cited. In short, there was a lack of high-level evidence designs, more prospectively, randomized controlled designs are required in the future.

The ERAS Study Group produced evidence-based protocols to promote perioperative management, which should be implemented by multidisciplinary teams, including surgeons, nurses, anesthesiologists, nutritionists, as well as patients, relatives, and caring members (6, 20). Though the ERAS protocol has been widely recognized around the world, there were still limited changes in most healthcare systems (6). In this study, the majority of the original articles were clinical research, followed by surveys, quality improvement studies, audits, cost-effectiveness analyses, and cross-sectional studies (21–25). We found that only quite a few studies had multidisciplinary interventions, however, most of them focused on elements of ERAS. The top research interests included “length of stay and early discharge after surgery,” “perioperative pain management,” “postoperative complications,” “implementation and compliance with ERAS.” In shorts, these most focused research interests may represent the hotspots in the ERAS research.

The ERAS was overwhelmingly implemented in colorectal surgeries (28%), followed by hip and knee arthroplasties, and cardiac surgeries. When classified by surgical disciplines, the department of General Surgery, Orthopedics, Gynecology, Cardiac Surgery, and Thoracic Surgery implemented the greatest number of ERAS protocols. It must be emphasized that the divide of surgical disciplines can differ in institutions. The Department of General Surgery implemented more than 50% ERAS protocols among all disciplines because most abdominal surgeries were included (except for urological, gynecological, and vascular surgeries). The disruption of gastrointestinal function in these surgeries required postoperative rehabilitation urgently. In terms of diseases, most of the ERAS protocols were primarily focused on malignancies, these patients were weak suffered more from surgical trauma. In detail, the ERAS protocols mainly served for colorectal cancers in colorectal surgeries (10, 17) kidney and liver diseases in the transplantation surgeries (26–28) osteoarthritis, degenerative spinal diseases, and hip fractures in orthopedics surgeries (12, 29, 30) coronary artery diseases in cardiac surgeries (31, 32). Fast-track anesthesia mainly focused on postoperative pain control and reduction of opioid use, early extubation, and reduction of postoperative complications (33–35).

Hotspots were further revealed by keywords that frequently occurred during a certain period, which were shown in Vosviewer, bursts were detected furtherly by Citespace. Not surprisingly, they exported very similar results. The most frequently occurring keywords “enhanced recovery after surgery” and “fast-track surgery” were not shown in the visualized map to better present other keywords. Though the ERAS concept was formally put forward in 2001, the keywords “ERAS” burst in 2017, before that, “fast-track surgery” was widely used. Research on cardiac surgery burst during 2000–2012, research on colorectal surgery peaked around 2016. In recent years, gastrectomy for bariatric surgery, cystectomy for bladder cancer, compliance with ERAS, and feasibility of ERAS in elderly patients gathered most research interests, which may represent the current trends in ERAS research. We hypothesize that these results were related to the increasing number of obesity, the aged tendency of the population, and the rising morbidity of malignancies. Barriers during the implementation of ERAS were multifactorial, not only from patients but from managers and practitioners (36). Recently, more studies focused on compliance or adherence to ERAS, sustainability of ERAS in community hospitals, and quality improvement programs (36–39).

This study has several limitations. First, the number of included articles was limited because we only retrieved the WOS database, research that followed ERAS protocols but described them as “accelerated recovery,” “rapid recovery,” “fast recovery” or “multimodal rehabilitation” can be dismissed by using the current search strategy. As was mentioned at the beginning of the article, the ERAS Study Group was established in 2001 and defined the concepts and goals of ERAS, before that, “fast-track recovery” had been used for several years, those studies were not included because of the limited numbers. Second, the contribution of countries and institutions can be influenced greatly by some productive authors, thus contributions from some influential authors and institutions may be underestimated. Furthermore, some influential authors, institutions, and most frequently occurring keywords can not be well-shown in the visualized map. Third, the distribution of countries was determined by corresponding authors, distribution of the authors and institutions was determined by full author lists, while the co-cited authors in the reference list were determined by the first authors, for studies that were performed by cooperation from different institutions and countries, the last corresponding author was regarded as the major contributor, which may lead to bias. Lastly, the level of evidence of the included studies is weak, indicating that most of the ERAS research was observational studies. This conclusion can be influenced by the included criteria and the grade of evidence level. Despite these facts, this bibliometric analysis presented a clear global distribution and hotspots of original ERAS in the recent 20 years.

## Conclusion

This study revealed the global status and trends in the field of ERAS research. Revealing the global states and hotspots can help researchers better understand the trends in ERAS research. The USA was the greatest contributor to ERAS research. Kehlet, H, was the most influential author in the field. Bariatric surgery, compliance with ERAS, and feasibility in the elderly represent the new trend of ERAS research. Most of the ERAS research had low evidence levels, studies with high-level evidence are still required in this field.

## Data Availability Statement

The original contributions presented in the study are included in the article/supplementary material, further inquiries can be directed to the corresponding author.

## Author Contributions

SS designed the research, retrieved the literature, and drafted the manuscript. TW screened the literature, graded the level of evidence, and analyzed the data. RW screened the literature and graded the level of evidence. QL performed the statistical analysis, modified the manuscript, and censored the article. MB designed the work, performed the analysis, performed the interpretation of data, and approved the version to be published. All authors contributed to the article and approved the submitted version.

## Conflict of Interest

The authors declare that the research was conducted in the absence of any commercial or financial relationships that could be construed as a potential conflict of interest.

## Publisher's Note

All claims expressed in this article are solely those of the authors and do not necessarily represent those of their affiliated organizations, or those of the publisher, the editors and the reviewers. Any product that may be evaluated in this article, or claim that may be made by its manufacturer, is not guaranteed or endorsed by the publisher.
